# Benefits of early ambulation within 24 h after total knee arthroplasty: a multicenter retrospective cohort study in China

**DOI:** 10.1186/s40779-021-00310-x

**Published:** 2021-03-05

**Authors:** Yi-Ting Lei, Jin-Wei Xie, Qiang Huang, Wei Huang, Fu-Xing Pei

**Affiliations:** 1 Department of Orthopedics, The First Affiliated Hospital of Chongqing Medical University, Chongqing, 400016 China; 2 Department of Orthopedics, West China Hospital, Sichuan University, 37# WainanGuoxue Road, Chengdu, 610041 China

**Keywords:** Total knee arthroplasty, Early ambulation, Length of stay, Costs, Deep venous thrombosis

## Abstract

**Background:**

Postoperative care has been evolving since the concept of enhanced recovery after surgery (ERAS) was introduced in China. This study aimed to evaluate the effects of early ambulation within 24 h after unilateral total knee arthroplasty (TKA) on postoperative rehabilitation and costs in a Chinese population.

**Methods:**

This cohort study of patients with knee osteoarthritis who had undergone TKA at 24 large teaching hospitals between January 2014 and November 2016 involved 2687 patients who began ambulating within 24 h (Group A) and 3761 who began ambulating later than 24 h (Group B). The outcome measurements, such as length of stay (LOS), total hospitalization costs, dynamic pain level, knee flexion range of motion (ROM), results of the 12-Item Short Form Survey (SF-12), incidence of thromboembolic events and other complications, were recorded and compared.

**Results:**

The early ambulation group (Group A) had a shorter LOS and lower hospitalization costs and pain levels than the late ambulation group (Group B). There was a favorable effect in enhancing ROM for patients in Group A compared with patients in Group B. In Group A, patients had significantly higher postoperative SF-12 scores than those in Group B. The incidence of deep venous thrombosis (DVT) and pulmonary infection was significantly lower in Group A than in Group B. The incidence of pulmonary embolism (PE) and other complications did not differ between the two groups.

**Conclusion:**

Early ambulation within 24 h after TKA was associated with reduced LOS, improved knee function, lower hospitalization costs and lower incidence of DVT and pulmonary infection in the Chinese population.

## Background

Total knee arthroplasty (TKA) is considered the most effective orthopedic procedure for the treatment of knee osteoarthritis and other knee diseases [1]. Since the concept of enhanced recovery after surgery (ERAS) was introduced in China, the spotlight has been placed on early activity intervention in postoperative rehabilitation for the orthopedic inpatient population [2]. A variety of regimens of perioperative intervention have been developed in an attempt to achieve enhanced recovery, including early ambulation, optimized pain and nausea management, blood management, and adjustment of the use of drains and catheters [3–5]. These efforts are always used in combination and have been shown to improve clinical outcomes. However, it is unclear what proportion of this can be attributed to early ambulation and what proportion is due to other interventions [6].

Early ambulation is a safe intervention with few contraindications and is thought to reduce the risks of thrombosis, urinary retention and pulmonary infection. To the best of our knowledge, there are few studies on the isolated contribution of early ambulation to improved clinical outcomes [7]. Chandrasekaran et al. [8] examined the isolated effect of early ambulation on the incidence of deep venous thrombosis. However, the sample size was relatively small (*n* = 100), and other outcomes, such as the duration of hospital stay and knee function, were not evaluated. In addition, the conclusion was more directed at the Western norm. Since lifestyle, diet, average body mass index (BMI) and nutrition status differ between ethnic groups, the optimal time to begin mobilization after TKA in the Asian population remains controversial and needs further investigation.

Therefore, we conducted a retrospective cohort study of patients with knee osteoarthritis who had undergone TKA to help clarify whether: (1) early ambulation within 24 h after TKA shortens length of stay (LOS), (2) early ambulation within 24 h is associated with better knee function and pain relief, (3) this regimen reduces total hospital costs, (4) this regimen decreases the incidence of thromboembolic events after TKA, and (5) this regimen increases the risk of treatment-related adverse effects.

## Methods

### Study population

The retrospective cohort study was a sub-study conducted within a large prospective observational study sponsored by the Chinese Health Ministry (201302007) on the efficacy and safety of perioperative management of total joint arthroplasty. The related data were collected from 24 large teaching hospitals in China. Patients with knee osteoarthritis who had undergone TKA at 24 large teaching hospitals between January 2014 and November 2016 were included in this study. Ethical approval was obtained from the West China Hospital of Sichuan University Biomedical Research Ethics Committee (2012–268).

The inclusion criteria for this substudy included patients aged 18 years and older, osteoarthritis as the primary diagnosis and indicator for primary TKA, and capable of providing informed consent. Those undergoing simultaneous bilateral TKA or staged bilateral TKA were excluded. In this study, patients who began ambulating within 24 h were identified as the early ambulation group (Group A), and those who began ambulating later than 24 h constituted the late ambulation group (Group B).

### Anesthesia and surgical procedures

A total of 4180 operations were performed under general anesthesia, and the remaining operations were performed under spinal, epidural or combined spinal-epidural anesthesia. All operations were performed by senior surgeons with a midline skin incision and medial parapatellar approach. A drainage catheter was applied in 4401 patients, and a tourniquet was applied in 4843 patients.

### Postoperative care protocol

The timing of first ambulation postoperatively mainly depended on the surgeons’ preferences. Early ambulation was defined as any partial or full weight-bearing activities (walking on the spot, bed-to-chair or bed-to-toilet) under the supervision of a physiotherapist within 24 h. Nonsteroidal anti-inflammatory drugs were used as the principal analgesic drugs according to each patient’s requirements.

A standardized protocol of thromboprophylaxis was well established among the 24 large joint reconstruction centers. Patients received 4000 U enoxaparin (Clexane; Sanofi-Aventis, France) or 10 mg rivaroxaban (Xarelto, Bayer, Germany) 6–8 h postoperatively, repeating at 24-h intervals for 14 d. Doppler ultrasound for lower extremities was performed routinely to detect deep venous thrombosis (DVT). Pulmonary embolism (PE) was investigated by clinical symptoms and contrast-enhanced chest CT scans.

### Outcome measurements

Patient demographic and preoperative characteristics were documented for comparison between the 2 groups. The LOS was recorded from the day of admission to discharge. Total hospitalization cost was defined as the total payment that the patients’ primary insurance carrier provided to the hospital.

Dynamic pain level was assessed using a visual analog scale (VAS, 0 means no pain, 10 means severe pain imaginable) and was conducted 72 h after surgery. The knee flexion range of motion (ROM) was assessed with a goniometer 72 h after the operation for each patient. The 12-Item Short Form Survey (SF-12) was conducted at the 4th week after the operation. Thromboembolic events and other complications were recorded during the inpatient hospital stay and 3-month follow-up period.

### Statistical analysis

All data analysis was performed using SPSS version 24 (SPSS, Inc., Chicago, IL, USA) software. Student’s t-test was performed to analyze the parametric samples, while the Wilcoxon Mann-Whiney *U* test was used for nonparametric data. For parametric samples, data are expressed as the means ± standard deviation. For nonparametric samples, data were expressed as M (Q_1_, Q_3_). Categorical data are expressed as *n* (%), and the Pearson chi-square test and Fisher’s exact test were performed. *P* < 0.05 was considered statistically significant for all comparisons.

## Results

### Patients’ demographics

**Fig. 1:**
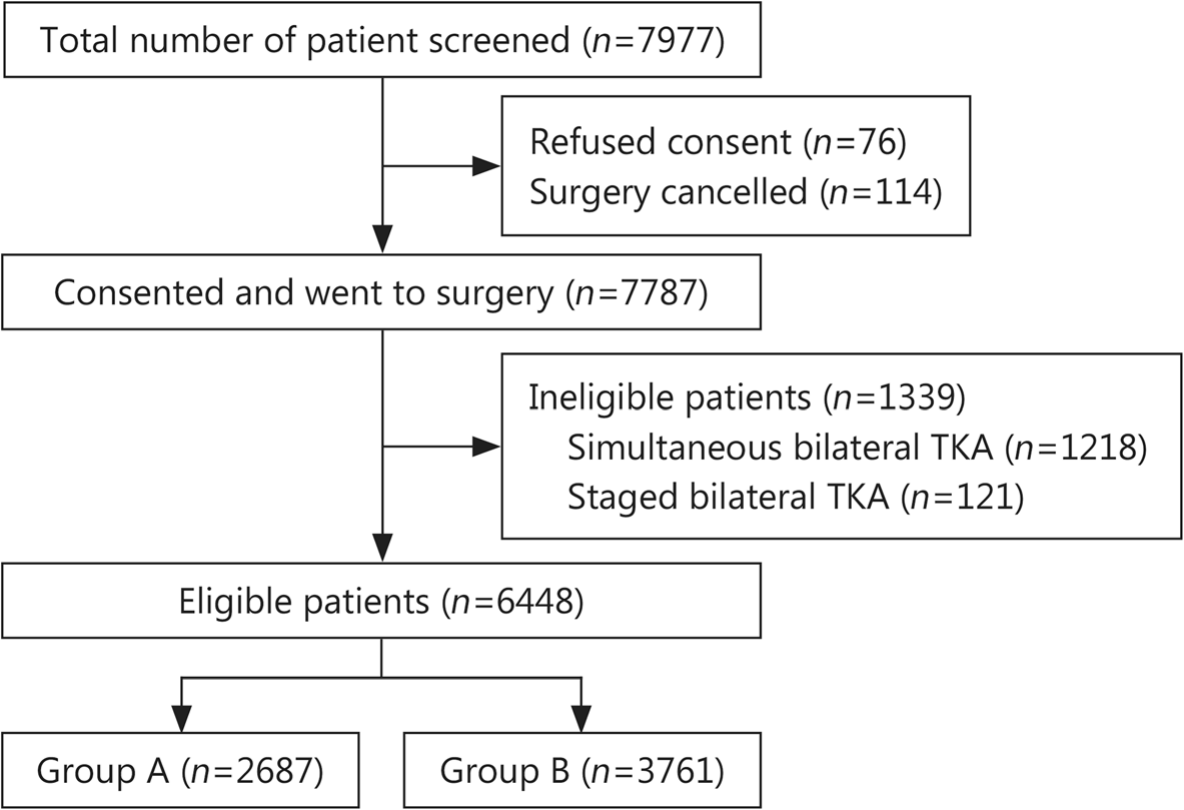
Flow diagram of the patients showing the study design

During the study period, TKA was performed on 7787 patients, of whom 1339 were excluded. The remaining 6448 patients were included in the final analysis, comprising 2687 who began ambulating within 24 h and 3761 who began ambulating later than 24 h (Fig. 1). These two groups had similar baseline demographic data (Table 1).

**Table 1:** Demographic data of the patients receiving unilateral TKA

Item	Group A (*n* = 2687)	Group B (*n* = 3761)	*P* value
Age [year, M (Q_1_, Q_3_)]	67 (61, 72)	67 (61, 73)	0.456
Gender [*n*(%)]			0.412
Male	582 (21.66)	847 (22.52)	
Female	2105 (78.34)	2914 (77.48)	
BMI [kg/m^2^, M (Q_1_, Q_3_)]	24.98 (22.60, 27.89)	25.39 (23.01, 27.78)	0.091
Hypertension [*n*(%)]	646 (24.04)	907 (24.12)	0.945
Diabetes [*n*(%)]	187 (6.96)	297 (7.90)	0.159
CHD [*n*(%)]	91 (3.38)	148 (3.94)	0.250
ASA class [*n*(%)]			0.281
1	860 (32.01)	1232 (32.76)	
2	1606 (59.77)	2175 (57.83)	
3	215 (8.00)	343 (9.12)	
4	6 (0.22)	11 (0.29)	
Anesthesia [*n*(%)]			0.451
General anesthesia	1762 (65.57)	2418 (64.29)	
Spinal anesthesia	175 (6.51)	272 (7.23)	
Epidural anesthesia	117 (4.35)	150 (3.99)	
CSEA	633 (23.56)	921 (24.49)	
Drainage [*n*(%)]	1852 (68.92)	2549 (67.77)	0.328
Tourniquet [*n*(%)]	2045 (76.11)	2798 (74.40)	0.117
Anticoagulation [*n*(%)]			0.283
Enoxaparin	1256 (46.74)	1431 (38.05)	
Rivaroxaban	1809 (67.32)	1952 (51.90)	
ROM (°, Mean ± SD)	84.88 ± 15.15	85.08 ± 14.88	0.592

TKA. Total knee arthroplasty; BMI. Body mass index; CHD. Coronary heart disease; ASA. American Society of Anesthesiologists; CSEA. Combined spinal epidural anesthesia; ROM. Range of motion; Group A. Early ambulation group; Group B. Late ambulation group

### LOS and total hospitalization costs

The LOS in Group A was significantly shorter than that in Group B (*P* < 0.001). In addition, total hospitalization costs were significantly lower in Group A than in Group B (*P* < 0.001, Table 2).

**Table 2:** Postoperative outcomes of the patients receiving unilateral TKA [M(Q_1_,Q_3_)]

Item	Group A (*n* = 2687)	Group B (*n* = 3761)	*P* value
LOS	10 (8, 12)	12 (8, 17)	< 0.001
THC	54,288.05 (48,733.93, 65,341.12)	58,115.62 (51,495.10, 69,325.00)	< 0.001
VAS score	2 (1, 3)	2 (1, 3)	< 0.001
ROM (°)	100 (97, 110)	100 (90, 110)	< 0.001
SF-12 score	49 (45, 50)	46 (42, 50)	< 0.001

TKA. Total knee arthroplasty; LOS. Length of stay; THC. Total hospitalization costs; VAS. Visual analog scale; ROM. Range of motion; SF-12. 12-Item Short Form Survey; Group A. Early ambulation group; Group B. Late ambulation group

### Postoperative pain level, ROM and SF-12 score

The VAS score at 72 h after surgery in Group A was significantly lower than that in Group B (*P* < 0.001). ROM improved significantly in both groups following TKA, and there was a favorable effect in enhancing ROM for patients in Group A compared with patients in Group B (*P* < 0.001). In Group A, patients had significantly higher postoperative SF-12 scores than those in Group B (*P* < 0.001, Table 2).

### Thrombosis and other complications

The occurrence of DVT in Group A was significantly lower than that in Group B (*P* = 0.008). In addition, the incidence of pulmonary infection in Group A was significantly lower than that in Group B (*P* = 0.031). The PE frequencies were 2 and 4 in groups A and B, respectively, and the difference was not statistically significant (*P* = 1.000). No significant differences were observed between the two groups in the incidence of other complications, such as falls, dislocations, periprosthetic joint infections, surgical wound infections or hematomas (Table 3).

**Table 3:** Complications of the patients receiving unilateral TKA [*n*(%)]

Item	Group A (*n* = 2687)	Group B (*n* = 3761)	*P* value
DVT	19 (0.71)	53 (1.41)	0.008
PE	2 (0.07)	4 (0.11)	1.000
Pulmonary infection	7 (0.26)	24 (0.64)	0.031
PJI	5 (0.19)	7 (0.19)	1.000
Superficial infection	11 (0.41)	13 (0.35)	0.679
Fall	21 (0.78)	26 (0.69)	0.675
Dislocations	2 (0.07)	3 (0.08)	1.000
Nerve damage	6 (0.22)	14 (0.37)	0.289
Periprosthetic fractures	4 (0.15)	5 (0.13)	1.000
Wound dehiscence	6 (0.22)	13 (0.35)	0.372
Hematoma	16 (0.60)	24 (0.64)	0.830

TKA. Total knee arthroplasty; DVT. Deep venous thrombosis; PE. Pulmonary embolism; PJI. Periprosthetic joint infection. Group A. Early ambulation group; Group B. Late ambulation group

## Discussion

This study aimed to describe the effects of early ambulation for TKA in terms of LOS, costs, knee function, pain, the incidence of complications and patient satisfaction. Early ambulation has traditionally been recommended for elective orthopedic surgery without adequate information regarding the ideal time to begin mobilization [8, 9]. Guerra et al. [10] systematically reviewed evidence from 5 randomized trials and suggested that early mobilization can be achieved within 24 h of operation. However, this review only focused on Australian, Spanish, Danish, British, and American patients [10], and no Asian patients were included in the review. Yue et al. [11] used 3-dimensional knee models to analyze knee anthropometry and suggested that there is a difference in size and shape between Asian and Caucasian knees. In addition, Iorio et al. [11, 12] showed that Asian patients had a significantly lower postoperative range of motion than Caucasian patients and suggested that racial morphologic differences might contribute to differences in outcome. Thus, the conclusion may be valid only for Western demographics, and the recommended timing of getting out of bed and walking after TKA in the Chinese population has not been decided.

To the best of our knowledge, this is the first study to define the isolated contribution of early ambulation to postoperative rehabilitation and costs in a Chinese population. With the aging of the population, the number of TKAs in China has increased year by year, and the associated cost has become a difficult problem for patients [13–15]. In China, a straightforward joint replacement cost is reported to be beyond the reach of many people in underdeveloped agricultural areas [16]. Molloy et al. [15] and Qi et al. [17] found that shorter hospital stays are usually associated with lower costs. Therefore, there is significant interest in identifying cost-effective strategies to shorten the recovery time of these patients. Early ambulation is a safe and feasible intervention requiring no additional costly equipment [18]. Pua et al. [7] reported that early ambulation can effectively shorten LOS by 0.69 d. Consistent with their findings, we demonstrated a significant reduction in LOS of almost 2.5 d in patients who began ambulating within 24 h after TKA. The benefit in reduced LOS was also achieved at hospitalization costs, with a reduction of nearly ¥4000, which could greatly abate the economic burden for patients.

ROM and content pain control are important factors for patient satisfaction after TKA [19–21]. Radulovic et al. [22] found that postoperative rehabilitation can effectively reduce swelling and improve ROM. Berend et al. [4] also indicated that rapid functional recovery after TKA may be more related to postoperative rehabilitation than to the size of the incision. Pearse et al. [18] reported that early mobilization was associated with fewer morphine requirements for postoperative pain relief. According to nationwide data, early ambulation within 24 h after TKA is associated with improved ROM and decreased postoperative pain, which is in accordance with previous studies. Although we cannot attribute the entire effect to early ambulation, the results might provide potential clinical relevance for this observation.

Health-related quality of life is widely recognized as a vital outcome for TKA [23]. In our study, we adopted the SF-12 to assess functional health and well-being from the patient’s point of view [23]. In the early ambulation group, patients had significantly higher postoperative SF-12 scores than patients in the late ambulation group, indicating that this active intervention could achieve a quick return to independence in daily activities, which might comprehensively reflect all the functional beneficial effects of the early ambulation regimen in our study.

DVT is a major complication of TKA, for which a variety of prophylactic interventions have been recommended [24, 25]. Buehler et al. [26] showed a lower incidence of DVT in patients who had immediate progressive weightbearing after operation compared to those who had delayed weightbearing. However, compared with Western countries, thrombotic events have traditionally been thought to be rare in Asian populations [27, 28], and the effect of early ambulation on thrombogenesis after TKA in the Chinese population is still inconclusive. In addition, considering the low incidence of these events, data from at least 2000 patients per arm would be needed to detect a 1% difference with 80% power and a 95% confidence interval [24]. Therefore, the sample size of their study was relatively small and might have been underpowered to identify such a difference. To provide additional evidence for this issue, 6448 patients were involved in our study, which might be more powerful for drawing conclusions than previous studies and demonstrated that early ambulation within 24 h after TKA was effective in decreasing the incidence of DVT and pulmonary infection, in line with previous literature [7, 8, 10].

Although many studies have proven the security of early ambulation [7, 9, 29], concerns about the complications related to early ambulation still hinder the wide adoption of this protocol in TKA. We therefore conducted this study to determine whether the treatment-related complications differed between groups after TKA. In this group of 2687 patients who ambulated within 24 h after TKA, we found a low occurrence of falls, dislocations, nerve damage and wound dehiscence, and the risk was not different between the two groups based on the timing of first ambulation. While further studies are still needed before the protocol can be recommended for general use, our results provide insight into assessing the safety of early ambulation in the Chinese population.

Several limitations should be noted. First and foremost, walking distance during the first postoperative ambulation was not recorded in this study, and this potential variability may have had an impact on the conclusions. However, with a large sample size, variability should be reduced. In addition, costs were considered only during an initial inpatient admission, and the costs of rehabilitation treatment after discharge were not shown in this study. As a developing country, the health policy and economic condition of China are quite different from those of Western developed countries [13]. Since most patients in China chose to go home after discharge [13], the additional costs after discharge might not change the results. Last but not least, despite surgeons’ preferences, other factors, such as the basic health condition of patients, could also influence the timing of first ambulation after TKA. However, there was no difference in baseline characteristics between the groups. In addition, previous studies have shown that more than 90% of patients with TKA were able to ambulate within 24 h after TKA [7, 18, 30], indicating that the percentage of patients who were allowed to ambulate early but failed to do so was small. Thus, we believe that this bias did not strongly affect the validity of the conclusions.

## Conclusions

Based on the findings, early ambulation within 24 h after TKA is suggested to have both positive clinical and economic consequences, and it seems to shorten LOS, reduce hospitalization costs, improve knee function, ameliorate postoperative pain and decrease the incidence of DVT and pulmonary infection in the Chinese population.

## Data Availability

The datasets supporting the results of this article are included within the article and its additional files.

## Abbreviations

ERAS: Enhanced recovery after surgery
TKA: Total knee arthroplasty
LOS: Length of stay
SF-12: 12-Item Short Form Survey
VAS: Visual analog scale
ROM: Range of motion
DVT: Deep venous thrombosis
PE: Pulmonary embolism
